# Association Study of Myosin Heavy Chain 15 Polymorphisms with Asthma Susceptibility in Chinese Han

**DOI:** 10.1155/2019/3805405

**Published:** 2019-02-18

**Authors:** Guo Chen, Lan Luo, Miao-Miao Zhang, Shou-Quan Wu, Yu Wang, Andrew J. Sandford, Jian-Qing He

**Affiliations:** ^1^Department of Respiratory and Critical Care Medicine, West China Hospital, Sichuan University, Chengdu, Sichuan, China; ^2^Department of Geriatrics, Sichuan Academy of Medical Sciences & Sichuan Provincial People's Hospital, Chengdu, Sichuan, China; ^3^Chinese Academy of Sciences Sichuan Translational Medicine Research Hospital, Chengdu, Sichuan, China; ^4^Centre for Heart Lung Innovation, University of British Columbia and St. Paul's Hospital, Vancouver, BC, Canada

## Abstract

**Background:**

The Myosin Heavy Chain 15 gene (*MYH15*) is expressed in the airway epithelium and variants in the gene have been associated with airway responsiveness. The aim of this study was to perform the first investigation of* MYH15 *polymorphisms in relation to asthma susceptibility.

**Methods:**

A total of 410 asthma patients and 418 controls from the Chinese Han population were enrolled in the study. Tag-single nucleotide polymorphisms were genotyped and associations between the polymorphisms and asthma risk were analyzed by logistic regression analysis adjusting for confounding factors. Dual-luciferase reporter gene analysis was performed to detect allele-dependent promoter activity of* MYH15 *variants in HEK293 cells.

**Results:**

The A allele of rs9288876 decreased risk of asthma (allelic model: OR=0.808, 95% CI: 0.658-0.993, additive model: OR=0.747, 95% CI: 0.588-0.947, dominant model: OR=0.693, 95% CI: 0.502-0.955). The G alleles of both rs7635009 and rs1454197 were associated with decreased risk of asthma under the additive model (OR=0.779, 95% CI: 0.618-0.981 and OR=0.756, 95% CI: 0.600-0.953, respectively). rs9288876 allele A was associated with higher luciferase activity than allele T (*P*<0.001). The luciferase activity of rs7635009 allele A was lower than allele G (*P*=0.001), while rs1454197 allele T had lower luciferase activity than allele G (*P*<0.001).

**Conclusion:**

This is the first study to report the association of* MYH15 *gene polymorphisms with asthma. Polymorphisms of rs9288876, rs7635009, and rs1454197 altered transcriptional regulation of* MYH15 *and may be functional variants conferring susceptibility to asthma. Further study with larger sample size in different ethnic populations is needed.

## 1. Introduction

Asthma is a chronic inflammatory airway disorder. As a global health problem, asthma affects a large number of people and results in significant morbidity. It has been estimated that 300 million people are suffering from asthma, the disease causes 250,000 deaths every year [1], and the number of patients has been predicted to increase to 400 million by 2025 [2]. The prevalence of asthma is increasing in China. Previous studies reported that the prevalence of asthma in children, aged from 0 to14 years, increased from 1.54% in 2000 to 3.02% in 2009 [3, 4]. The prevalence of asthma among individuals aged >14 years was 1.24% in the China Asthma and Risk factors Epidemiologic (CARE) survey [5], which was the latest and largest investigation in China. It is estimated that there are approximately 30 million asthmatic patients in China [5]. The risk factors for asthma include female sex, age, smoking, first-degree relatives suffering from asthma or allergic rhinitis, eczema, or gastroesophageal reflux disease [5].

The incidence of asthma varies and ranges from 1% to 18% in different countries [6]. Asthma is a heterogeneous disease caused by the interactions of genetic and environmental factors. As the first barrier between the human airway and the environment, the airway epithelium is an important controller of the immune, inflammatory, and regenerative reactions to environmental triggers of asthma, and its structural and functional impairment is considered to be an initial factor in the development of an asthma [7]. Polymorphisms of several genes expressed in the airway epithelium were reported to be involved in the pathophysiologic mechanisms underlying asthma. Variants in a disintegrin and metalloprotease 33 (*ADAM33*) and protocadherin-1 (*PCDH1*) were reported to be involved in airway hyperresponsiveness and airway remodeling [8–11]. Thymic stromal lymphopoietin (*TSLP*) played an important role in the initiation of allergic inflammation and provided protection against asthma by inducing differentiation of airway epithelium cells and increasing the repair response to airway epithelium injury [12, 13]. An antibody against* TSLP *was an effective treatment for asthma. Orosomucoid 1-like 3 (*ORMDL3*) and gasdermin B (*GSDMB*) polymorphisms were associated with childhood asthma [14]. Cystatin SN (*CST1*) may differentiate asthmatics with exercise-induced bronchoconstriction from those without this phenotype [15, 16].

Myosin heavy chain 15 (*MYH15*) located in chromosome 3q13.13 with 44 exons was previously reported to be expressed in extraocular muscles [17]. The protein encoded by* MYH15 *is a slow-twitch myosin mainly associated with muscle contraction. Myosins are a large family of motor proteins involved in adenosine triphosphate hydrolysis, actin binding, and kinetic energy transduction. Recently,* MYH15 *polymorphisms were associated with airway responsiveness (p=5.41x10^−8^) and the protein was shown to be expressed in the airway epithelium and alveolar macrophages of patients with chronic obstructive pulmonary disease (COPD) [18]. However, to date there have been no studies of the association between* MYH15 *polymorphisms and asthma. Therefore, the aim of this study was to investigate the association of polymorphisms of* MYH15 *with asthma susceptibility in the Chinese Han population.

## 2. Materials and Methods

### 2.1. Study Population

A total of 410 asthmatic patients and 418 controls, who were unrelated Chinese Han individuals, were enrolled from the West China Hospital of Sichuan University in this case control study. Clinical information was collected from the outpatient electronic medical records, questionnaire and telephone follow-up, including age, sex, height, weight, smoking history, the age of asthma onset, and lung function. A total of 5 ml of peripheral venous blood was collected from every participant in Ethylene Diamine Tetraacetic Acid (EDTA) anticoagulant tubes and was stored in a −80°C freezer. The diagnosis of asthma was according to the criteria of the Global Strategy for Asthma Management and Prevention. Exclusion criteria for the cases were a respiratory disease other than asthma, tumor, immune diseases, and the use of hormones or immunosuppressive drugs. The controls were healthy volunteers collected from the physical examination center in the West China Hospital. Informed consent was obtained from every participant included in the study. The study was approved by the ethical committee of the West China Hospital of Sichuan University in 2013 (Protocol no. 23).

### 2.2. Tag-SNP Selection and Genotyping

**Figure 1:**
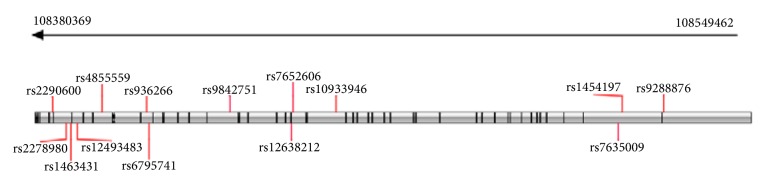
The locations of tag-SNPs in the* MYH15* with the exons. The grey bar represented the* MYH15* gene. The black lines inside the grey bar from right to left represented the exons 1-44, respectively.

Single nucleotide polymorphisms (SNPs) with minor allele frequency (MAF) ≥ 0.10, located within the region 3000 base pairs upstream to 300 base pairs downstream of* MYH15, *were downloaded from the Han Chinese in Beijing database of the Genome Variation Server 147 (http://gvs.gs.washington.edu/GVS/) which enabled rapid access to human genotype data found in dbSNP and provided tools for analysis of genotype data. The final selections were 14 tag-SNPs, including rs12638212, rs6795741, rs9842751, rs7635009, rs2278980, rs1463431, rs4855559, rs9288876, rs936266, rs7652606, rs2290600, rs1454197, rs12493483, and rs10933946 (Figure 1). Genomic DNA was extracted from the peripheral venous blood samples using a genomic DNA purification kit (Axygen Scientific Inc, Union City, CA, USA). Tag- SNPs were genotyped by Genesky Bio-Tech Co., Ltd. (http://geneskybiotech.com/index.html) using the SNPscanTM multiplex SNP genotyping technique based on double ligation and multiplex fluorescence PCR [19]. The probes and primers were designed using the SpectroDESIGNER software (Sequenom, Sequenom Inc., San Diego, CA, USA).

Approximately 5% of random samples were repeatedly genotyped with a concordance rate of 100%. The JASPAR database (http://jaspar.genereg.net/) and F-SNP database (http://compbio.cs.queensu.ca/F-SNP/) were used to predict the function of asthma susceptibility SNPs.

### 2.3. Functional Analysis of Asthma-Associated* MYH15 *Polymorphisms

The dual-luciferase reporter gene system was used to detect allele-dependent promoter activity of* MYH15 *polymorphisms. HEK293 cells were transfected with the firefly luciferase reporter plasmid pGL3-promoter (Promega, USA) under the control of the* MYH15 *region containing each allele of the asthma-associated SNPs (rs9288876, rs7635009, and rs1454197). The pRL-CMV renilla luciferase reporter plasmid (Promega, USA) was cotransfected for normalization of transfection efficiency, and dual-luciferase reporter assays were read 24 hours later with a GloMax 96 Microplate Luminometer. The results were described as relative fold changes in the constructed vector compared with the pGL3-promoter vector.

### 2.4. Data Analysis

Hardy-Weinberg equilibrium (HWE) among the controls was tested using the Chi-squared test. Continuous and categorical variables were analyzed using the Mann–Whitney U test and Pearson's Chi-squared test, respectively. Genotype distributions under different genetic models were examined by multivariate logistic regression analysis, adjusting for age, sex, body mass index (BMI), and smoking history. The false discovery rate was calculated by R to account for multiple testing. Linkage disequilibrium and haplotype analyses were calculated using the SHEsis online software (http://analysis.bio-x.cn) [20, 21]. The multifactor dimensionality reduction software (MDR3.0.2) [22] was used to identify the interactions of* MYH15 *tag-SNPs on asthma susceptibility. Statistical tests were performed using the Statistical Package for the Social Sciences (SPSS, SPSS Inc., Chicago, IL, USA), version 17.0. A p value <0.050 was considered to be statistically significant.

## 3. Results

### 3.1. Subject Characteristics

The study was composed of 159 males and 251 females in the case group (mean age: 44.02±13.77 years) and 162 males and 256 females in the control group (mean age: 44.09±13.75 years) from the Chinese Han population. The mean age of asthma onset in the cases was 33.69±14.26 years. There were no significant differences in age, sex, BMI, and smoking history between the two groups (Table 1).

### 3.2. SNP, Haplotype, and MDR Analysis

**Figure 2:**
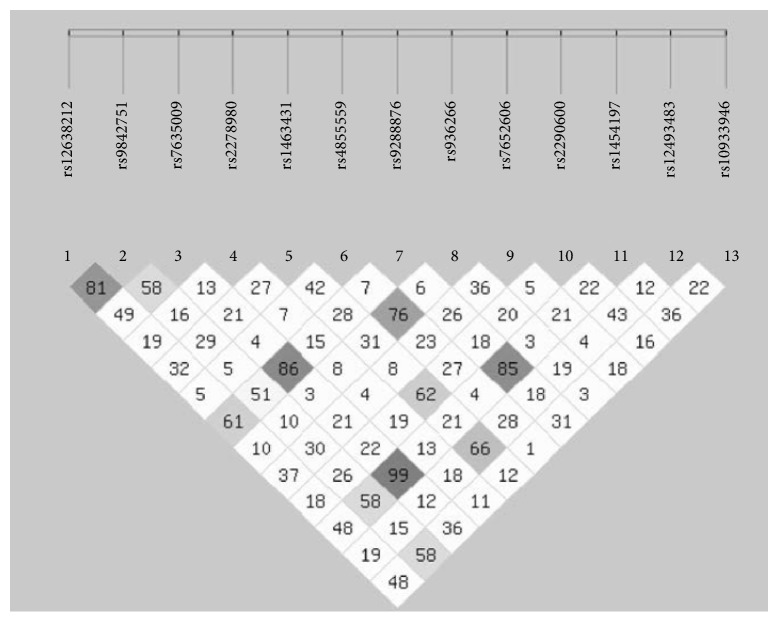
The analysis of linkage disequilibrium of tag-SNPs in* MYH15*. The linkage disequilibrium of the 13 tag-SNPs in* MYH15 *calculated by the SHEsis software (http://analysis.bio-x.cn).

The basic characteristics of the tag-SNPs were listed in the Table 2. As the distribution in the controls was not in Hardy-Weinberg equilibrium, rs6795741 was excluded from the study. After adjusting for confounding factors including age, sex, BMI, and smoking history, rs9288876 allele A was associated with decreased risk of asthma (allelic model: P=0.042, OR=0.808, 95% CI: 0.658-0.993, additive model: P=0.016, OR=0.747, 95% CI: 0.588-0.947, dominant model: P=0.025, OR=0.693, 95% CI: 0.502-0.955). The rs7635009 AG/GG genotypes showed an association with decreased risk of asthma under the dominant model (P=0.041, OR=0.712, 95% CI: 0.514-0.986). As the number of copies of the rs7635009 G allele increased, the risk of asthma decreased under the additive model (P=0.033, OR=0.779, 95% CI: 0.618- 0.981). The rs1454197 TG/GG genotypes decreased risk of asthma under the dominant model (P=0.018, OR=0.673, 95% CI: 0.485-0.933). As the number of copies of the rs1454197 G allele increased, the risk of asthma decreased under the additive model (P=0.018, OR=0.756, 95% CI: 0.600-0.953). However, no significant association was found after adjustment for multiple testing (Table 3). No significant difference was observed in the* MYH15 *haplotype frequencies between the case and control groups (P>0.05) (Table 4). There was high linkage disequilibrium between rs9288876, rs7635009, and rs1454197 (Figure 2). No interaction of the above 3 tag- SNPs was found in the MDR analysis (Table 5).

### 3.3. Stratification Analysis

We performed stratification analysis for age, gender, BMI, and smoking history, respectively. Significant associations are shown in the supplementary document (Table S1). The rs9288876 AA/TA genotypes showed an association with decreased risk of asthma under the dominant model among males (OR=0.557, 95% CI: 0.332-0.933). There was an inverse association between the number of copies of rs9288876 A allele and the risk of asthma under the additive model among nonsmokers (OR=0.765, 95% CI: 0.588-0.996). Additionally, an increased rs9288876 A allele copy number was associated with decreased risk of asthma in the population with normal BMI (OR=0.738, 95% CI: 0.550-0.989) and in those aged less than 60 years (allelic model, OR=0. 786, 95% CI: 0.629-0.982; additive model, OR=0.597, 95% CI: 0.385-0.925; dominant model, OR=0.463, 95% CI: 0.260-0.825), respectively. Both the rs2290600 CC genotype and increased copy number of the C allele were associated with increased the risk of asthma among smokers (recessive model, OR=2.984, 95% CI: 1.194-7.459; additive model, OR=1.860, 95% CI: 1.102-3.138).

The rs1454197 G allele was associated with decreased risk of asthma in nonsmokers (allelic model, OR=0.777, 95% CI: 0.603-1.000; additive model, OR=0.747, 95% CI: 0.576-0.967) and participants who were less than 60 years old (allelic model, OR=0.805, 95% CI: 0.648-1.000; additive model, OR=0.664, 95% CI: 0.441-0.999; dominant model, OR=0.528, 95% CI: 0.296-0.944). An increased number of rs12493483 G allele was associated with increased risk of asthma under the additive model among nonsmokers (OR=1.301, 95% CI: 1.016-1.665). The rs7635009 GG/AG genotype and increased copy number of G alleles were associated with decreased risk of asthma in the population aged less than 60 years (additive model, OR=0.664, 95% CI: 0.441-0.999; dominant model, OR=0.528, 95% CI: 0.296-0.944).

The rs4855559 allele T was associated with increased risk of asthma in the population with BMI<18.5 (OR=2.556, 95% CI: 1.037-6.297) but with decreased risk of asthma in the overweight population (24≤BMI<28) (OR=0.486, 95% CI: 0.286-0.825). The rs7652606 allele G was associated with increased risk of asthma in the population with BMI<18.5 (OR=2.850, 95% CI: 1.022-7.944) and rs936266 allele T with decreased risk in the overweight population (24≤BMI<28) (OR=0.521, 95% CI: 0.313-0.868). There was an inverse association between the number of copies of rs12638212 G allele and the risk of asthma in the population with normal BMI (OR=0.555, 95% CI: 0.321-0.959). The rs2278980 TT/CT genotypes and increased number of the T allele were associated with decreased risk of asthma in the population with normal BMI (dominant model, OR=0.330, 95% CI: 0.148-0.737; additive model, OR=0.416, 95% CI: 0.204-0.849).

### 3.4. Functional Analysis

**Figure 3:**
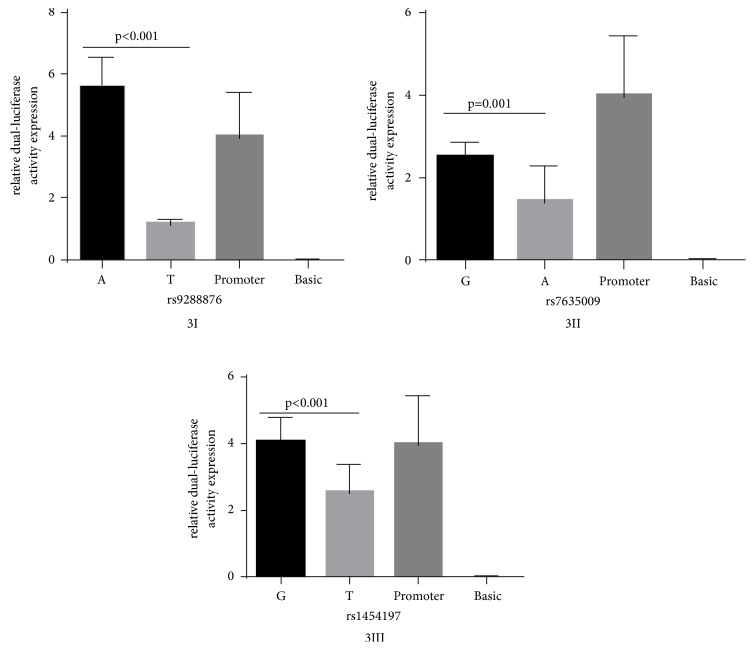
The allele-dependent dual-luciferase activity expression of* MYH15 *in HEK293 cells. Relative luciferase activity was measured in HEK293T cells transfected with firefly luciferase reporter plasmid pGL3-promoter containing each allele of the asthma-associated SNPs (rs9288876, rs7635009, and rs1454197). The pRL-CMV renilla luciferase reporter plasmid was cotransfected for normalization of transfection efficiency. The results are shown as relative fold changes in the constructed vector compared with the pGL3-promoter vector. Figure 3 I: the allele-dependent dual-luciferase activity expression of rs9288876 (A versus T: p<0.001, t-test). Figure 3 II: the allele-dependent dual-luciferase activity expression of rs7635009 (G versus A: p=0.001, t-test). Figure 3 III: the allele-dependent dual-luciferase activity expression of rs1454197 (G versus T: p<0.001, t-test). A: PGL3-promoter-A, T: PGL3-promoter-T, G: PGL3-promoter-G, Promoter: PGL3-promoter, and Basic: PGL3-basic.

All tag-SNPs were analyzed for their influence on the function of MYH15 with the JASPAR and F-SNP databases. The 5′ flanking region SNP rs9288876 as well as rs7635009 and rs1454197 in intron 1 were predicted to be possible functional SNPs with regulatory effects on gene transcription. Therefore, dual-luciferase reporter gene analysis was performed for these SNPs. The rs9288876 allele A increased the luciferase activity while the allele T reduced the luciferase activity (P<0.001) (Figure 3 I). Both the rs7635009 allele A and G reduced the luciferase activity, while the former had stronger ability to reduce the luciferase activity than the latter (P=0.001) (Figure 3 II). The rs1454197allele T significantly reduced the luciferase activity than allele G (P<0.001) (Figure 3 III).

## 4. Discussion

As a complex disease, asthma is caused by the interactions of multiple susceptibility genes, epigenetic regulatory mechanisms, and environmental risk factors [23, 24]. The airway epithelium in asthma patients exhibits an abnormal structure and function which is more susceptible to stimulation by allergen, virus, and environmental pollutants [25]. Although genetic studies have detected many susceptibility genes for asthma, efforts are needed to identify more specific functional genes which may be potential targets for effective asthma intervention and treatment in the future.* MYH15 *was previously reported to be expressed in extraocular muscles [17]. Its expression in other tissues and function were unknown. Neary et al. [26] reported that the T allele of rs29016420 in* MYH15 *was associated with decreased bovine pulmonary arterial pressure. Bare et al. [27] found that rs3900940 in* MYH15 *was associated with risk of coronary heart disease in a white population. May et al. [28] demonstrated that* MYH15 *was associated with risk of ischemic stroke in white participants, but not in black participants. In this work, we are the first to demonstrate that* MYH15 *polymorphisms may be associated with asthma in the Chinese Han population.

In 2015, an association study performed by Hansel et al. [18] reported that polymorphisms of* MYH15 *on chromosome 3 were related to the development of airway responsiveness in European American smokers with COPD, who were from the Lung Health Study (LHS). The researchers performed expression quantitative trait loci (eQTL) analyses in lung tissues to show cis-eQTL association with* MYH15 *gene expression and immunohistochemistry to identify MYH15 protein expressed in the airway epithelium, vascular endothelium, and inflammatory cells, which are all involved in the variation in airway responsiveness. Although a large-scale genome-wide association study (GWAS) reported by Moffatt et al. [29] in 2010 did not demonstrate any gene showing genome-wide significant association with asthma in chromosome 3, the possible effect of* MYH15 *in asthma could not be definitely ruled out. On the one hand, Moffatt et al. [29] used random-effects pooled analysis to test for the association in the overall population from 23 studies, but none of these studies included individuals from Chinese Han population. On the other hand, the association between* MYH15 *and respiratory diseases may not have been investigated in candidate gene studies, as its expression in the airway epithelium was not known prior to the study by Hansel et al.

As airway hyperresponsiveness is one of the representative pathophysiological mechanisms of asthma, we hypothesized that polymorphisms of* MYH15 *would be associated with asthma and this is what we demonstrated in the Chinese Han in the present study. Firstly, we found that the rs9288876 allele A, rs7635009 allele G, and rs1454197 allele G showed a protective effect against asthma in the association study. Those SNPs were different from the GWAS SNPs associated with airway responsiveness in the LHS, a multicenter randomized trial of bronchodilators in COPD, possibly due to the different diseases under study and ethnic background of the participants. The MAFs of rs2399233, rs1456711, and rs7618314 on* MYH15 *reported in the European Americans from the LHS were 0.0875, 0.0898, and 0.083, respectively, while these SNPs were not polymorphic in the Chinese Han population in 1000 Genomes. Secondly, we applied the dual-luciferase reporter gene method and found that rs9288876, rs7635009, and rs1454197 are potential functional variants due to the alterations in transcriptional regulation in the promoter of* MYH15*. These observations may be explained by the locations of the variants which are the 5′ flanking region and intron 1 of the gene. It was interesting that the luciferase activity of each mutant allele (rs9288876 allele A, rs7635009 allele G, and rs1454197 allele G) was higher than the wild-type allele. There was a tendency that each allele associated with decreased susceptibility to asthma demonstrated higher luciferase expression.

Therefore we speculate that the above genetic variants are involved in asthma by the transcriptional regulation of* MYH15*. However, the detailed mechanism underlying our results is unknown.

This study was the first attempt to investigate the association between* MYH15 *and asthma in the Chinese population and to identify the functional SNPs. Our data may be valuable for research into the genetic mechanisms underlying other diseases and may be of clinical benefit in the future, including being used for genetic diagnosis and even in the treatment of asthma.

As asthma is attributed to multiple factors, the effect of each polymorphism on asthma susceptibility is weak. Although the results after the multiple testing correction were negative, it is still possible that* MYH15 *variants are related to asthma in the Chinese Han population in light of the positive functional analysis. Therefore, this issue needs further study with larger sample sizes and replication in different ethnic populations. The limitations of the study were as follows: firstly, only smoking was considered as an environmental factor, while other factors including air pollution and viral infection were not taken into account. Secondly, the functional test only referred to the allele-dependent transcriptional regulation in the promoter. The specific transcriptional factors involved in the regulation of the mRNA expression in different genotypes need further research.

In conclusion,* MYH15 *gene polymorphisms may be associated with asthma in the Chinese Han population. The* MYH15 *rs9288876 allele A, rs7635009 allele G, and rs1454197 allele G may reduce the risk of asthma. The rs9288876, rs7635009, and rs1454197 were potential functional variants due to the transcriptional regulation activity in the promoter of* MYH15*. This issue needs further study with larger sample sizes in different ethnic populations.

## Figures and Tables

**Table 1:** Characteristics of cases and controls.

Characteristic	Cases (N=410)	Controls (N=418)	*P *value
Age (years)	44.02 ± 13.77	44.09 ± 13.75	0.944
Male n (%)	159 (38.78)	162 (38.76)	0.994
BMI (kg/m^2^)	23.06 ± 3.19	22.95 ± 3.34	0.634
Smoking history n (%)	76/403(18.86)	55/262(20.99)	0.58
Age of asthma onset (years)	33.69 ± 14.26	-	-
FEV1% predicted	81.36 ± 23.99	-	-
FEV1/FVC %	71.38 ± 14.85	-	-
FVC % predicted	95.63 ± 10.18	-	-
Serum IgE (IU/ml)	219.68 ± 412.59	-	-

Values are means ± standard deviation (SD) and absolute numbers (valid percentages). FEV1: forced expiratory volume in one second. FVC: forced vital capacity. IgE: immunoglobulin E. BMI: body mass index.

**Table 2:** Characteristics of the tag-SNPs in *MYH15*.

chromosome	SNPs	SNPs location	Function	Alleles^*∗*^	MAF^*∗*^
3	rs9288876	108529575	upstream variant 2kb	T>A	0.34
3	rs1454197	108519798	Intron 1	T>G	0.38
3	rs7635009	108518915	Intron 1	A>G	0.36
3	rs10933946	108451830	Intron 22	T>A	0.34
3	rs7652606	108441492	Intron 23	A>G	0.26
3	rs12638212	108441130	Missense Exon 24	A>G	0.45
3	rs9842751	108426596	Intron 28	T>C	0.48
3	rs936266	108406767	Intron 33	C>T	0.30
3	rs6795741	108407444	Intron 33	G>A	0.46
3	rs4855559	108396189	Intron 36	G>T	0.24
3	rs12493483	108390670	Intron 38	G>A	0.45
3	rs2278980	108387820	Intron 39	C>T	0.17
3	rs1463431	108388857	Intron 39	C>T	0.43
3	rs2290600	108384580	Intron 40	A>C	0.40

*∗*Han Chinese in Beijing, China, from the database of Genome Variation Server 147 (http://gvs.gs.washington.edu/GVS/).

**Table 3:** *MYH15 *polymorphisms in cases and controls.

SNPs	Case group N	Control group N	Genetic model ^b^	OR (95% CI)	*P* ^*∗*^	*P* ^#^
rs12638212 (A>G)	410	418	Add	0.902 (0.721-1.129)	0.367	0.682
AA	149 (0.363)	143 (0.342)	Dom	0.836 (0.598-1.170)	0.297	0.644
AG	187 (0.456)	197 (0.471)	All	1.0589 (0.871-0.287)	0.571	0.874
GG	74 (0.180)	78 (0.187)	Rec	0.925 (0.614-1.395)	0.711	0.938
rs9842751 (T>C)	410	418	Add	0.887 (0.710-1.108)	0.293	0.635
TT	125 (0.305)	122 (0.292)	Dom	0.863 (0.606-1.227)	0.411	0.763
TC	197 (0.480)	198 (0.474)	All	0.936 (0.772-1.136)	0.503	0.874
CC	88 (0.215)	98 (0.234)	Rec	0.839 (0.576-1.224)	0.363	0.642
rs7635009 (A>G)	410	418	Add	0.779 (0.618-0.981)	0.033	0.143
AA	182 (0.444)	160 (0.383)	Dom	0.712 (0.514-0.986)	0.041	0.178
AG	178 (0.434)	198 (0.474)	All	1.197 (0.979-1.463)	0.080	0.347
GG	50 (0.122)	60 (0.144)	Rec	0.731 (0.463-1.155)	0.180	0.642
rs2278980 (C>T)	410	418	Add	0.958 (0.720-1.274)	0.767	0.860
CC	277 (0.676)	276 (0.660)	Dom	0.925 (0.660-1.295)	0.649	0.921
CT	117 (0.285)	131 (0.313)	All	1.009 (0.786-1.295)	0.945	0.955
TT	16 (0.039)	11 (0.026)	Rec	1.119 (0.482-2.598)	0.794	0.938
rs1463431 (C>T)	410	418	Add	1.000 (0.806-1.239)	0.997	0.997
CC	136 (0.332)	135 (0.323)	Dom	1.018 (0.727-1.425)	0.918	0.950
CT	182 (0.444)	194 (0.464)	All	0.994 (0.819-1.207)	0.955	0.955
TT	92 (0.224)	89 (0.213)	Rec	0.976 (0.667-1.428)	0.902	0.948
rs4855559 (G>T)	410	418	Add	1.033 (0.809-1.319)	0.794	0.860
GG	240 (0.585)	238 (0.569)	Dom	0.941 (0.682-1.298)	0.712	0.921
GT	130 (0.317)	148 (0.354)	All	0.987 (0.791-1.231)	0.907	0.955
TT	40 (0.098)	32 (0.077)	Rec	1.455 (0.807-2.624)	0.213	0.642
rs9288876 (T>A)	410	418	Add	0.747 (0.588-0.947)	0.016	0.117
TT	200 (0.488)	176 (0.421)	Dom	0.693 (0.502-0.955)	0.025	0.163
TA	171 (0.417)	191 (0.457)	All	0.808 (0.658-0.993)	0.042	0.347
AA	39 (0.095)	51 (0.122)	Rec	0.669 (0.405-1.103)	0.115	0.642
rs936266 (C>T)	410	418	Add	1. 058 (0.833-1.343)	0.645	0.860
CC	225 (0.549)	228 (0.545)	Dom	0.990 (0.719-1.362)	0.950	0.950
CT	141 (0.344)	156 (0.373)	All	0.267 (0.761-1.172)	0.605	0.874
TT	44 (0.107)	34 (0.081)	Rec	1.379 (0.792-2.399)	0.256	0.642
rs7652606 (A>G)	410	418	Add	0.932 (0.707-1.229)	0.620	0.860
AA	261 (0.637)	253 (0.605)	Dom	0.902 (0.650-1.253)	0.538	0.874
AG	131 (0.320)	148 (0.354)	All	1.088 (0.859-1.378)	0.484	0.874
GG	18 (0.044)	17 (0.041)	Rec	1.026 (0.468-2.250)	0.948	0.948
rs2290600 (A>C)	410	418	Add	1.045 (0.837-1.304)	0.700	0.860
AA	105 (0.256)	106 (0.254)	Dom	1.053 (0.735-1.509)	0.779	0.921
AC	202 (0.493)	210 (0.502)	All	0.991 (0.817-1.201)	0.924	0.955
CC	103 (0.251)	102 (0.244)	Rec	1.068 (0.739-1.544)	0.725	0.938
rs1454197 (T>G)	410	418	Add	0.756 (0.600-0.953)	0.018	0.117
TT	182 (0.444)	157 (0.376)	Dom	0.673 (0.485-0.933)	0.018	0.163
TG	178 (0.434)	201 (0.481)	All	0.823 (0.673-1.006)	0.057	0.347
GG	50 (0.122)	60 (0.144)	Rec	0.731 (0.463-1.155)	0.180	0.642
rs12493483 (A>G)	410	418	Add	1.155 (0.929-1.436)	0.196	0.598
AA	125 (0.305)	130 (0.311)	Dom	1.240 (0.884-1.738)	0.212	0.644
AG	190 (0.463)	206 (0.493)	All	0.919 (0.758-1.116)	0.395	0.874
GG	95 (0.232)	82 (0.196)	Rec	1.188 (0.808-1.747)	0.382	0.642
rs10933946 (T>A)	410	418	Add	1.154 (0.913-1.460)	0.230	0.598
TT	135 (0.329)	149 (0.356)	Dom	1.200 (0.860-1.675)	0.284	0.644
TA	207 (0.505)	202 (0.483)	All	1.070 (0.878-1.302)	0.498	0.874
AA	68 (0.166)	67 (0.160)	Rec	1.213 (0.777-1.893)	0.395	0.642

OR, 95% CI: Odds Ratio, 95% Confidence Interval. N: number of cases and controls. Values are absolute numbers (decimals) and OR, 95% CI. *∗*Adjusted for sex, age, body mass index, and smoking history with logistic regression. ^#^Corrected by false discovery rate. ^b^All: allelic model.

Add: additive model; Dom: dominant model; Rec: recessive model. SNPs: single nucleotide polymorphisms.

**Table 4:** Haplotype analysis of *MYH15 *in cases and controls.

Haplotype	Case group N	Control group N	x^2^	*P∗*	OR (95% CI)
A T A C C G T C A A T A T	49.19 (0.060)	53.03 (0.063)	0.078	0.781	0.944 [0.629-1.417]
A T A C C G T C A A T G A	245.93 (0.300)	235.09 (0.281)	0.925	0.336	1.121 [0.888-1.414]
A T A C T T T T A C T A A	51.89 (0.063)	53.54 (0.064)	0.002	0.962	0.990 [0.664-1.477]
G C A T T G T C A C T A T	27.97 (0.034)	18.59 (0.022)	2.195	0.138	1.566 [0.861-2.848]
G C G C C G A C G A G G T	14.27 (0.017)	25.60 (0.031)	3.076	0.079	0.559 [0.290-1.080]
G C G C T T A T G C G A T	95.58 (0.117)	102.81 (0.123)	0.151	0.697	0.941 [0.694-1.277]
G C G T T G A C A C G A T	98.94 (0.121)	108.36 (0.130)	0.295	0.587	0.920 [0.682-1.242]
others^†^	236.23 (0.288)	238.98 (28.6)	-	-	-

For each haplotype, alleles were arranged in order of rs12638212-rs9842751-rs7635009- rs2278980-rs1463431-rs4855559-rs9288876-rs936266-rs7652606-rs2290600-rs1454197-rs12493483-rs10933946. OR, 95% CI: Odds Ratio, 95% Confidence Interval. N: number of haplotype in case and control group. x^2^: value tested by Pearson's Chi-squared test. *∗*Adjusted for sex, age, BMI, and smoking history with logistic regression. ^†^The lowest frequency threshold (LFT) < 0.03 was pooled in this part.

**Table 5:** The MDR analysis of the interaction of rs7635009, rs9288876, and rs1454197.

Model	Testing balanced accuracy	Cross-validation consistency	*P∗*
rs1454197	0.5247	6/10	0.2080
rs9288876, rs1454197	0.5273	6/10	0.1650
rs7635009, rs9288876, rs1454197	0.5347	10/10	0.0730 - 0.0740

*∗P *value for testing balanced accuracy using 1,000-fold permutation test.

## Data Availability

All data used to support the findings of this study are available from the corresponding author upon request (Proessor Jian-Qing He, email: jianqhe@gmail.com).

## Supplementary Materials

Supplementary Materials We performed stratification analysis for age, gender, BMI, and smoking history, respectively. In order to make the table succinct, only positive associations were shown in Table S1.
