# Supplementary material for: Association Study of Myosin Heavy Chain 15 Polymorphisms with Asthma Susceptibility in Chinese Han
Source: Biomed Res Int. 2019 Feb 18;2019:3805405. doi: 10.1155/2019/3805405 (PMC6398074; doi:10.1155/2019/3805405)
Supplement: Supplementary Materials — We performed stratification analysis for age, gender, BMI, and smoking history, respectively. In order to make the table succinct, only positive associations were shown in Table S1. [file 3805405.f1.docx]

**Supplementary tables:**

We performed stratification analysis for age, gender, BMI and smoking history, respectively. In order to make the table succinct, only positive associations were shown in the table S1.

Table S1 Stratification analysis of *MYH15* polymorphisms in cases and controls

| SNPs | Stratification factors | Asthma | Control | Genetic models | *P* | OR (95%CI) |
| --- | --- | --- | --- | --- | --- | --- |
|  |  | N (%) | N (%) |  |  |  |
| rs9288876(T>A) | male | 159 | 162 | All | 0.082 | 0.738(0.523-1.040) |
| TT |  | 81(0.509) | 56(0.394) | Add | 0.052 | 0.678(0.459-1.003) |
| TA |  | 64(0.403) | 71(0.500) | **Dom** | **0.026** | **0.557(0.332-0.933)** |
| AA |  | 14(0.088) | 15(0.106) | Rec | 0.581 | 0.786(0.334-1.847) |
| rs9288876(T>A) | non-smoking | 334 | 207 | All | 0.079 | 0.793(0.612-1.028） |
| TT |  | 159(0.476) | 87(0.420) | **Add** | **0.047** | 0.765(0.588-0.996) |
| TA |  | 144(0.431) | 91(0.440) | Dom | 0.117 | 0.752(0.526-1.074) |
| AA |  | 31(0.093) | 29(0.140) | Rec | 0.080 | 0.612(0.352-1.061) |
| rs9288876(T>A)  TT  TA  AA | BMI 2 | 245  118(0.482)  103(0.420)  24(0.098) | 166  64(0.386)  79(0.476)  23(0.139) | **All**  Add  Dom  Rec | **0.042**  0.171  0.067  0.990 | **0.738（0.550-0.989）**  0.678（0.389-1.182）  0.495 (0.233-1.051)  1.007(0.308-3.293) |
| rs9288876(T>A)  TT  TA  AA  rs2290600(A>C) | age<60y  smoking | 346  168(0.486)  147(0.425)  31(0.090)  69 | 362  150(0.414)  167(0.461)  45(0.124)  55 | **All**  **Add**  **Dom**  Rec  All | **0.034**  **0.021**  **0.009**  0.498  0.053 | **0.786(0.629-0.982)**  **0.597(0.385-0.925)**  **0.463(0.260-0.825)**  0.720(0.279-1.861)  0.609(0.367-1.009） |
| AA |  | 17(0.246) | 19(0.345) | **Add** | **0.020** | 1.860(1.102-3.138) |
| AC |  | 28(0.406) | 25(0.455) | Dom | 0.120 | 1.925(0.843-4.392) |
| CC |  | 24(0.348) | 11(0.200) | **Rec** | **0.019** | 2.984(1.194-7.459) |
| rs1454197(T>G) | non-smoking | 334 | 207 | **All** | **0.05** | 0.777(0.603-1.000) |
| TT |  | 143(0.428) | 75(0.362) | **Add** | **0.027** | 0.747(0.576-0.967） |
| TG |  | 152(0.455) | 97(0.469) | Dom | 0.062 | 0.706(0.490-1.018） |
| TT |  | 39(0.117) | 35(0.169) | Rec | 0.081 | 0.639(0.387-1.056） |
| rs1454197(T>G)  TT  TG  GG  rs12493483(A>G) | age<60y  non-smoking | 346  154(0.445)  151(0.436)  41(0.118)  334 | 362  133(0.367)  178(0.492)  51(0.141)  207 | **All**  **Add**  **Dom**  Rec  All | **0.050**  **0.049**  **0.031**  0.384  0.058 | **0.805(0.648-1.000)**  **0.664(0.441-0.999)**  **0.528(0.296-0.944)**  0.695(0.307-1.577)  0.787(0.614-1.009） |
| AA |  | 100(0.299) | 77(0.372) | **Add** | **0.037** | **1.301(1.016-1.665)** |
| AG |  | 156(0.467) | 91(0.440) | Dom | 0.068 | 1.414(0.974-2.053) |
| GG |  | 78(0.234) | 39(0.188) | Rec | 0.107 | 1.445(0.924-2.261) |
| rs7635009(A>G) | age<60y | 346 | 362 | All | 0.064 | 1.228 (0.988-1.526) |
| AA  AG  GG |  | 154(0.445)  151(0.436)  41(0.118) | 135(0.373)  176(0.486)  51(0.141) | **Add**  **Dom**  Rec | **0.049**  **0.031**  0.384 | **0.664(0.441-0.999)**  **0.528(0.296-0.944)**  0.695(0.307-1.577) |
| rs12638212(A>G) | BMI 2 | 245 | 166 | All | 0.228 | 1.188(0.897-1.573) |
| AA |  | 85(0.347) | 51(0.307) | **Add** | **0.035** | **0.555(0.321-0.959)** |
| AG |  | 115(0.469) | 77(0.464) | Dom | 0.126 | 0.524(0.229-1.200) |
| GG |  | 45(0.184) | 38(0.229) | Rec | 0.053 | 0.396(0.155-1.010) |
| rs2278980(C>T)  CC  CT  TT | BMI 2 | 245  163(0.665)  73(0.298)  9 (0.037) | 166  103(0.620)  56(0.337)  7(0.042) | All  **Add**  **Dom**  Rec | 0.373  **0.016**  **0.007**  0.828 | 1.171(0.827-1.660)  **0.416(0.204-0.849)**  **0.330(0.148-0.737)**  0.794(0.098-6.407) |
| rs4855559(G>T)  GG  GT  TT  rs4855559(G>T)  GG  GT  TT | BMI 1  BMI 3 | 22  14(0.636)  7(0.318)  1(0.045)  107  64(0.598)  29(0.271)  14(0.131) | 29  10(0.345)  15(0.517)  4(0.138)  80  59(0.738)  18(0.225)  3(0.037) | **All**  Add  Dom  Rec  **All**  Add  Dom  Rec | **0.039**  0.076  0.078  0.998  **0.007**  0.401  0.489  0.447 | **2.556（1.037-6.297）**  0.066(0.003-1.326)  0.059(0.003-1.370)  0.000(0.000- )  **0.486（0.286-0.825）**  1.313(0.695-2.480)  1.372(0.561-3.356)  1.728(0.423-7.065) |
| rs936266(C>T)  CC  CT  TT | BMI 3 | 107  63(0.589)  28(0.262)  16(0.150) | 80  56(0.700)  21(0.263)  3(0.037) | **All**  Add  Dom  Rec | **0.012**  0.337  0.389  0.447 | **0.521（0.313-0.868）**  1.370(0.721-2.604)  1.484(0.605-3.640)  1.728(0.423-7.065) |
| rs7652606(A>G)  AA  AG  GG | BMI 1 | 22  16(0.727)  6(0.273)  0(0.000) | 29  14(0.483)  12(0.414)  3(0.103) | **All**  Add  Dom  Rec | **0.040**  0.273  0.440  0.999 | **2.850（1.022-7.944）**  0.374(0.065-2.170)  0.414(0.044-3.887)  0.000(0.000- ) |

OR, 95% CI: Odds Ratio, 95% Confidence Interval. N: number of cases and controls. Values are absolute numbers (valid percentages) and OR, 95% CI. SNPs: single nucleotide polymorphisms. All: allelic model; Add: additive model; Dom: dominant model; Rec: recessive model. non-smoking: without smoking history. BMI: body mass index. BMI1: BMI<18.5. BMI2: 18.5≤BMI<24. BMI3: 24≤BMI<28. BMI4: BMI≤28.
